# Maternal microchimerism in the livers of patients with Biliary atresia

**DOI:** 10.1186/1471-230X-4-14

**Published:** 2004-07-31

**Authors:** David L Suskind, Philip Rosenthal, Melvin B Heyman, Denice Kong, Greg Magrane, Lee-Ann Baxter-Lowe, Marcus O Muench

**Affiliations:** 1 Department of Pediatrics, University of California, San Francisco, USA; 2 Immunogenetics, University of California, San Francisco, USA; 3 Laboratory Medicine, University of California, San Francisco, USA; 4 Children's Hospital, University of Washington, Seattle, USA

## Abstract

**Background:**

Biliary atresia (BA) is a neonatal cholestatic disease of unknown etiology. It is the leading cause of liver transplantation in children. Many similarities exist between BA and graft versus host disease suggesting engraftment of maternal cells during gestation could result in immune responses that lead to BA. The aim of this study was to determine the presence and extent of maternal microchimerism (MM) in the livers of infants with BA.

**Methods:**

Using fluorescent in situ hybridization (FISH), 11 male BA & 4 male neonatal hepatitis (NH) livers, which served as controls, were analyzed for X and Y-chromosomes. To further investigate MM in BA, 3 patients with BA, and their mothers, were HLA typed. Using immunohistochemical stains, the BA livers were examined for MM. Four additional BA livers underwent analysis by polymerase chain reaction (PCR) for evidence of MM.

**Results:**

By FISH, 8 BA and 2 NH livers were interpretable. Seven of eight BA specimens showed evidence of MM. The number of maternal cells ranged from 2–4 maternal cells per biopsy slide. Neither NH specimen showed evidence of MM. In addition, immunohistochemical stains confirmed evidence of MM. Using PCR, a range of 1–142 copies of maternal DNA per 25,000 copies of patients DNA was found.

**Conclusions:**

Maternal microchimerism is present in the livers of patients with BA and may contribute to the pathogenesis of BA.

## Background

Biliary atresia (BA) is a cholestatic disease of infancy characterized by the destruction of the biliary tree. [1-3] Both the intra-and extra-hepatic biliary ducts demonstrate evidence of a progressive destruction. This results in cholestasis, hepatic fibrosis and eventually cirrhosis. BA is associated with significant morbidity and mortality. Although the incidence of BA is 1 in 10,000 to 14,000 live births, [4,5] it accounts for over 40% of the neonatal cholestatic liver disease in Europe and the United States. [6] Prior to the hepatic portoenterostomy and liver transplantation, infants with BA had less than a 10% survival at 3 years of life and almost 100% mortality at 7 years of life. [7] In the United States, it is the leading cause of pediatric liver transplantation. [8] The etiology of biliary atresia remains unknown.

Many hypotheses on the etiology of BA exist. Two leading hypothesis are that BA occurs as a result of ductal plate malformations occurring during development of the liver, or as a result of an immune mediated process triggered by yet to be determined stimulus. [9] As more knowledge accrues about the histology and immunologic characteristics of BA, the more it appears to be a progressive immune mediated process.

Graft versus host disease (GVHD), seen after allogeneic hematopoietic stem cell transplantation, is an immune mediated process triggered by the transfer of alloimmune cells from a donor to a host. GVHD shares many similarities with BA. Both BA and GVHD are characterized by a lymphocytic infiltration around the portal triad and damage to the biliary ducts. [10] The predominant lymphocytes in both disease are CD4^+ ^T – helper (Th) 1 cells. [11-14] As with BA, in GVHD there is also an increase in cell adhesion molecules and human leukocyte antigen (HLA) class II markers. [11]

Given the similarities between BA and GVHD, we hypothesized that a contributing etiology of BA could be an alloimmune reaction such as in GVHD triggered by maternal microchimerism. Maternal microchimerism occurs when a small number of maternal cells are transferred to the offspring during pregnancy. This is known to occur in up to 40% of normal pregnancies. [15,16] In addition, a clinical precedent of maternal microchimerism causing hepatic GVHD in children with severe combined immune deficiency (SCID) exists. [17] The aim of this study was to determine the presence and extent of maternal microchimerism in the livers of infants with BA.

## Methods

This study was performed with the approval of the Committee of Human Research at USCF (H9048-20247-01). Cases of BA were identified through a data search in the Anatomic Pathology CoPath system by diagnosis or during patient medical visits to the UCSF Division of Pediatric Gastroenterology, Hepatology, and Nutrition. An explanation of the study was given to each study candidate. Written consent was obtained from each study participant.

### Fluorescent in situ hybridization (FISH) of male BA livers

We modified a previously reported method of FISH, [18] using X and Y-chromosomes probes. Y-chromosomes were stained with a green fluorescent dye, fluorescein isothiocyanate (FITC). X chromosomes were stained with a red fluorescent dye, cyanine 3 (Cy-3). Nuclear material was stained with a blue fluorescent dye 4", 6"-diamidino-2-phenylindole (DAPI). After initial digestion and staining of the liver specimens, we examined the slides for evidence of female cells, depicted by two red signals, i.e. X chromosomes, with no green signal within the blue nuclear material. Slides were analyzed in a blinded fashion.

### HLA typing

Patients with BA and their mothers were HLA typed. The HLA typing HLA-A, -B, and -DRB1 alleles of the child and mother were determined by sequence specific PCR (SSP) (Pel-Freez Clinical Systems, LLC^®^, Brown Deer, WI, USA).

### Immunohistochemistry

Frozen tissues sections of BA patient's livers (5 μm) were fixed in acetone for 10 minutes at 4°C then washed in PBS (5 minutes centrifugation × 3). Sections were blocked with Protein Block (Dako, Carpinteria, CA, USA) for thirty minutes at room temperature. Sections were then incubated with anti-HLA-B14 mouse monoclonal antibody (US Biological, Swampscott, Mass. USA) then washed in PBS. Sections were incubated in goat anti-mouse conjugated with FITC for 30 minutes at room temperature. Sections were rewashed and then counterstained with DAPI. Slides were then examined using a fluorescent microscope.

### Kinetic Polymerase Chain Reaction (kPCR)

Using the HLA types of the patients with BA and their mothers, and a previously reported method of kPCR, evidence of maternal DNA was explored within the liver biopsy specimens of explanted BA livers. [18] The minor modifications to the kinetic PCR protocol included use of iCyler from BioRad (Hercules, Ca, USA) for amplification.

## Results

### Detection of female cells in male BA liver biopsies

**Figure 1:**
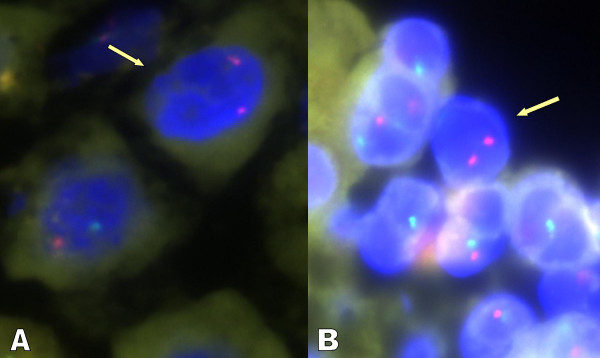
A male cell, depicted by a red signal, the X chromosome, and green signal, a Y chromosome within the blue nuclear material, can be seen in both male BA liver specimens (A&B). In the same specimen, one can also see a female cell, depicted by two red signals, both X-chromosomes with no green signal within the blue nuclear material (arrow).

Using FISH, we examined 11 male BA liver biopsies and 4 male NH liver biopsies. After initial digestion and staining of the liver specimens, we were able to interpret 8 of the BA and 2 NH liver biopsy specimens (Table 1). Three BA and 2 NH biopsies underwent poor digestion and had no discernable signal. Figure 1 shows a liver from a male infant with BA. A male cell, depicted by a red signal, the X chromosome, and green signal, a Y chromosome within the blue nuclear material, clearly can be seen (A). In the same specimen, one can also see a female cell, depicted by two red signals, both X-chromosomes with no green signal within the blue nuclear material (B). 7/8 BA specimens had evidence of maternal microchimerism. The number of maternal cells, per biopsy slide, was 2 – 4 cells. Neither of the NH specimens had evidence of maternal microchimerism.

**Table 1:** Overview of maternal microchimerism in patients with BA and NH

Diagnosis	Chimerism	Method	# of Maternal cells/DNA per slide (FISH) or per 25,000 copies of patient's DNA (kPCR)
BA	Present	FISH	2 maternal cells per slide
BA	Present	FISH	2 maternal cells per slide
BA	Present	FISH	4 maternal cells per slide
BA	Present	FISH	2 maternal cells per slide
BA	Present	FISH+immunokistochemistry	3 maternal cells per slide
BA	Not interpretable	FISH	---
BA	Present	FISH	2 maternal cells per slide
BA	Present	FISH	3 maternal cells per slide
BA	Not interpretable	FISH	---
BA	Not interpretable	FISH	---
BA	None detected	FISH	0 maternal cells per slide
BA	Present	kPCR	3 copies of maternal DNA per 25,000 copies of patient's DNA
BA	Present	kPCR	1 copy of maternal DNA per 25,000 copies of patient's DNA
BA	Present	kPCR	10 copies of maternal DNA per 25,000 copies of patient's DNA
BA	Present	kPCR	142 copies of maternal DNA per 25,000 copies of patient's DNA
NH	None detected	FISH	0 maternal cells per slide
NH	None detected	FISH	0 maternal cells per slide
NH	Not interpretable	FISH	---
NH	Not interpretable	FISH	---

### Detection of maternal HLA antigen among BA liver samples

**Figure 2:**
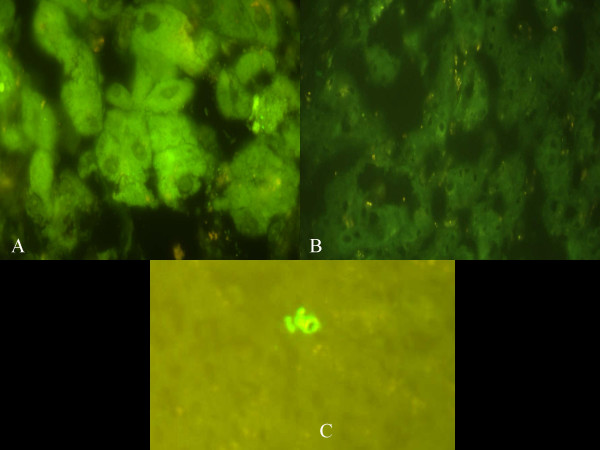
Using the HLA marker B14, we examined the BA livers for evidence of maternal microchimerism using immunohistochemistry techniques. An HLA-B14 positive patient served as our positive control (A). Specimen B, our negative control, was a patient who was, along with his mother, negative for HLA-B14. Specimen C, our test specimen, was HLA-B14 negative, with an HLA-B14+ mother. Specimen A (positive control) has a bright signal for HLA-B14 while there is only background staining for HLA-B14 in specimen B (negative control). Specimen C, the test specimen, has isolated scattered bright signals suggesting maternal microchimerism.

To further investigate our findings, three patients with BA and their mothers were HLA typed. Using the HLA marker B14, we examined the BA livers for evidence of maternal microchimerism using immunohistochemistry techniques. An HLA-B14 positive patient served as our positive control (Figure 2). Specimen B, our negative control, was a patient who was, along with his mother, negative for HLA-B14. Specimen C, our test specimen, was HLA-B14^-^, with an HLA-B14^+ ^mother. Specimen A (positive control) has a bright signal for HLA-B14 while there is only background staining for HLA-B14 in specimen B (negative control). Specimen C, the test specimen, has isolated scattered bright signals suggesting maternal microchimerism.

### Detection of maternal DNA by kPCR

**Figure 3:**
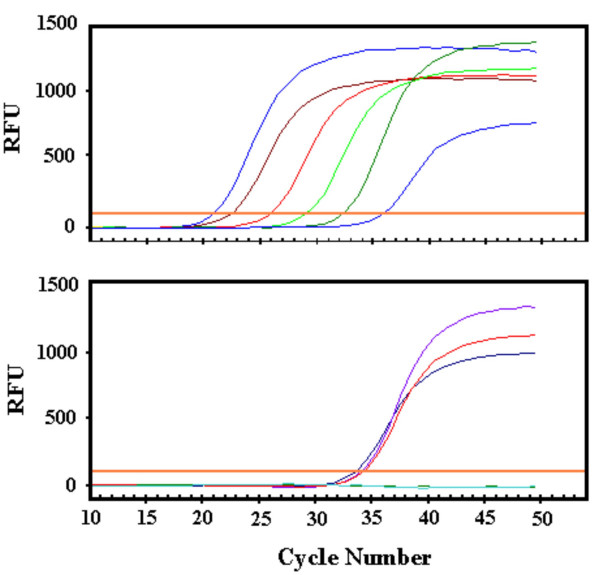
Representative kinetic PCR (kPCR) data for maternal HLA-B40 in BA liver sample. SYBR Green fluorescence is plotted on the y-axis as a function of amplification cycle number on the x-axis. The upper panels depicts results for standard dilutions of HLA-B40 [left to right curves: 25000, 10000, 1000, 100,10, and 1 genomic equivalence (gEq)]. A single copy of template produces fluorescent signal after approximately 36 cycles of amplification. The lower panel shows results from three liver replicates and two negative (no-template) controls. The three liver replicates have a positive signals at 2–5 gEq, while the negative controls remained negative through 50 cycles.

Finally, using polymerase chain reaction, in conjunction with the HLA typing of patients and mothers, we were able to affirm that maternal microchimerism occurs in the BA livers. An additional four fresh frozen liver specimens of BA infants, obtained at the time of transplantation, were examined for maternal DNA. All the livers had evidence of maternal microchimerism, with the range of 1 to 142 copies of maternal DNA detected per 25000 copies of patients' DNA. Figure 3 is a representative kPCR for maternal HLA-B40 in the second liver sample.

In all eleven of twelve patients with BA had evidence of maternal microchimerism by FISH or kinetic PCR, while neither of the two neonatal hepatitis patients had evidence of maternal microchimerism (p = 0.03).

## Discussion

BA is a progressive cholestatic disease of infancy, which is characterized by a mild to moderate lymphocytic infiltration within the extra and intrahepatic bile ducts. It is plausible, that maternal microchimerism may play a role in the etio-pathogenesis of BA by causing an alloimmune reaction given the similarities between BA and GVHD. We have shown that maternal microchimerism is present within the livers of patients with BA.

Maternal microchimerism occurs when maternal cells reside in the body of offspring. Maternal-fetal lymphocytic transfer is known to occur during pregnancy starting as early as the tenth week of gestation and continuing up to delivery. [19] The number of cells that traverse the placenta increases throughout this period. Schroder et al [15] demonstrated that approximately 1/10 infants has about 0.07% maternal lymphocytes at the time of birth and more recent findings by Lo et al. [16] have indicated the presence of maternal cells in over 40% of fetal blood samples.

Disease has been shown to occur in mothers who have had fetal microchimerism, and children/adults who have had maternal microchimerism. Children who have immunodeficiencies are at greater risk for graft verses host disease owing to engraftment of maternal lymphocytes. In both SCID and DiGeorge syndrome, maternal microchimerism and GVHD have been well described. In a study by Susanne Müller et al., 121 patients with SCID were evaluated for maternal microchimerism using HLA typing. Maternal cells were found in 48 patients, with 19 patients showing signs of GVHD. GVHD manifested itself in the skin and in the liver. [20] There have been numerous case reports showing GVHD in infancy of patients with DiGeorge. Manifestations of this disease include skin, gastrointestinal tract and liver involvement. [21]

The histologic and immunologic similarities of BA and GVHD are striking. The site of damage in BA and GVHD after bone marrow transplantation is the same. In each, lymphocytes congregate around the bile ducts. The damage occurs to both the intra-and extrahepatic biliary tracts. In a mouse model of acute GVHD, Nonomura et al. showed that transfer of allogeneic cells set along minor HLA mismatch can cause damage to both the intra and extrahepatic biliary ducts. Interestingly, the timeframe in this mouse model of acute GVHD, in terms of lymphocytic infiltration and fibrosis, appear similar to that of BA. [14] Initially, a peak of lymphocytes around the bile ducts occurs about 2 weeks after transfer of allogeneic cells from donor to host mouse; as the lymphocytic infiltration subsides, liver fibrosis increases. This correlates with disease progression in BA, where initial diagnostic biopsies of BA livers usually show a larger lymphocytic infiltration, and less fibrosis, as compared to biopsies done later, i.e. at the time of liver transplantation. [22]

The lymphocytic infiltrates in both BA and GVHD are predominantly CD4^+ ^T lymphocytes. The genes in BA livers showed differential lymphocytic function, with activation of osteopontin, a regulator of cell-mediated immunity in Th 1 cells, and interferon gamma. [23] Similarly, in GVHD, the predominant leukocyte is the CD4^+ ^T lymphocyte. Although a variety of complex mechanisms are involved in causing the end organ damage in GVHD, evidence exists showing similarities in effector mechanisms. In a SCID mouse model of acute GVHD, an increase in interferon-gamma secretion with a synchronized increase in activated Th cells was seen during acute GVHD. [24] The inflammatory responses in both BA and GVHD are also associated with increased expression of adhesion molecules such as CD54, and increased class II HLA markers, such as HLA-DR. HLA-DR and CD54, in both BA and GVHD, are seen predominantly around the bile ducts. [11,25,26]

Maternal microchimerism is not a rare occurrence in healthy individuals, but is usually not associated with disease. The high occurrence of maternal microchimerism in healthy individuals has been suggested to have a tolergenic effect that may contribute to long-term microchimerism. [27]Additionally, in utero transplantation of haploidentical cells does not always lead to immune tolerance [28] and may lead to immune sensitization. Thus, the immunological consequences of the migration of maternal cells to the fetus appear to be variable. In most cases the maternal cells are likely to be cleared by the host's immune system or they may escape destruction by the immune system leading to engraftment. Engraftment of maternal immune cells in the fetal biliary tract may result in an immune reaction against the host cholangiocytes. This is what we believe may occur in infants with BA. Alternatively, engrafted maternal cells in the fetus may subsequently be rejected by the immune system of the offspring, leading to destruction of the biliary tree.

Maternal microchimerism occurs in the livers of patients with BA. Given the previously well-described relationship between microchimerism and GVHD, and the similarities between BA and GVHD, these findings indicate a potential etio-pathogenisis for BA. Further investigations into the role of maternal microchimerism in BA are warranted.

## Competing interests

None declared.

## Author's contributions

DLS conceived of the hypothesis and contributed to the design and coordination of the study; he also drafted the manuscript. PR and MBH participated in the design of the study and manuscript preparation. DK carried out the kPCR and HLA typing analyses. GM performed the FISH analyses. LBL carried out the kPCR and HLA typing analyses and participated in the study design. MOM participated on the study design and coordination, immunohistochemistry stains and the manuscript preparation.

## Pre-publication history

The pre-publication history for this paper can be accessed here:


